# Spinal Cord Parenchyma Vascular Redistribution Underlies Hemodynamic and Neurophysiological Changes at Dynamic Neck Positions in Cervical Spondylotic Myelopathy

**DOI:** 10.3389/fnana.2021.729482

**Published:** 2021-11-23

**Authors:** Zhengran Yu, Xing Cheng, Jiacheng Chen, Zhong Huang, Shaofu He, Hao Hu, Sixiong Lin, Zhiyuan Zou, Fangli Huang, Bolin Chen, Yong Wan, Xinsheng Peng, Xuenong Zou

**Affiliations:** ^1^Guangdong Provincial Key Laboratory of Orthopaedics and Traumatology, Department of Spine Surgery, The First Affiliated Hospital, Sun Yat-sen University, Guangzhou, China; ^2^Institute of Neuroanatomy and Cell Biology, Hannover Medical School, Hanover, Germany; ^3^Department of Radiology, The First Affiliated Hospital, Sun Yat-sen University, Guangzhou, China; ^4^Department of Orthopedic, The First Affiliated Hospital of Nanchang University, Nanchang, China

**Keywords:** cervical spondylotic myelopathy, dynamic neck positions, motor evoked potentials, somatosensory evoked potentials, spinal cord perfusion, vascular redistribution

## Abstract

Cervical spondylotic myelopathy (CSM) is a degenerative condition of the spine that caused by static and dynamic compression of the spinal cord. However, the mechanisms of motor and somatosensory conduction, as well as pathophysiological changes at dynamic neck positions remain unclear. This study aims to investigate the interplay between neurophysiological and hemodynamic responses at dynamic neck positions in the CSM condition, and the pathological basis behind. We first demonstrated that CSM patients had more severe dynamic motor evoked potentials (DMEPs) deteriorations upon neck flexion than upon extension, while their dynamic somatosensory evoked potentials (DSSEPs) deteriorated to a similar degree upon extension and flexion. We therefore generated a CSM rat model which developed similar neurophysiological characteristics within a 4-week compression period. At 4 weeks-post-injury, these rats presented decreased spinal cord blood flow (SCBF) and oxygen saturation (SO_2_) at the compression site, especially upon cervical flexion. The dynamic change of DMEPs was significantly correlated with the change in SCBF from neutral to flexion, suggesting they were more sensitive to ischemia compared to DSSEPs. We further demonstrated significant vascular redistribution in the spinal cord parenchyma, caused by angiogenesis mainly concentrated in the anterior part of the compressed site. In addition, the comparative ratio of vascular densities at the anterior and posterior parts of the cord was significantly correlated with the perfusion decrease at neck flexion. This exploratory study revealed that the motor and somatosensory conductive functions of the cervical cord changed differently at dynamic neck positions in CSM conditions. Compared with somatosensory conduction, the motor conductive function of the cervical cord suffered more severe deteriorations upon cervical flexion, which could partly be attributed to its higher susceptibility to spinal cord ischemia. The uneven angiogenesis and vascular distribution in the spinal cord parenchyma might underlie the transient ischemia of the cord at flexion.

## Introduction

Cervical spondylotic myelopathy is a degenerative condition of the spine that leads to static and dynamic compression of the spinal cord (Karadimas et al., 2013). It has been proposed that dynamic injury may occur through instability (Jiang et al., 2011), an increase in the range of motion (Matsunaga et al., 2002), and minor trauma in pre-existing degenerative cervical myelopathy (Fengbin et al., 2013). Numerous clinical studies have reported the impacts of dynamic neck positions on CSM. On the morphological front, Muhle et al. (1998a) used dynamic MRI to demonstrate that the prevalence of spinal stenosis was increased upon flexion and extension compared to that upon a neutral position. Neurophysiologically, CSM patients’ DSSEPs have been shown to deteriorate significantly upon extension and flexion (Morishita et al., 2013; Qi et al., 2020). We further revealed that the percent changes in DSSEP amplitude at dynamic neck positions were related to preoperative radiographic characteristics, such as the presence of cervical segmental instability, compression degrees, and patterns of intramedullary T2WI hyperintensity (Yu et al., 2020). Pathophysiological changes in the spinal cord at dynamic positions were demonstrated by cadaveric studies, in which the lateral columns and the anterior horns were deformed by mechanical stress produced by spondylotic bars during flexion (Breig et al., 1966). Notably, the tissue of the spinal cord is highly vascularized and extremely sensitive to hypoxia induced by static or dynamic compression. The flexion position causes anterior compression of the cord, where the ASA traverses. Thus, it has also been suggested that ischemia of the spinal cord as a result of neck flexion can cause neurological deteriorations and cervical myelopathies (Hirayama and Tokumaru, 2000; Fujimoto et al., 2002; Restuccia et al., 2003). However, due to the lack of appropriate animal studies, the mechanisms behind the changes in neurological functions at dynamic neck positions are still not clear.

In recent years, numerous attempts have been made to establish CSM in various animal models, such as tumor induction (Izumida, 1995), the use of penetrating hydrogels (Yang et al., 2015), or urethane polymers (Kurokawa et al., 2011), or spinal hyperostotic mice (twy/twy) (Hirai et al., 2013), and plastic screw implantation (Kanchiku et al., 2001). Among these approaches, hydrogels and polymers are expected to be applicable materials that lead to chronically progressive injury. Previously, we successfully established a CSM rat model using a water-absorbable polyurethane polymer sheet (Long et al., 2013), which provided the foundation for this study. We found that various changes, including decreased microvascular density (Long et al., 2014; Cheng et al., 2015), varying degrees of ischemia-hypoxia (Long et al., 2012; Cheng et al., 2019), blood-spinal cord barrier (BSCB) disruption (Long et al., 2015; Cheng et al., 2019), and neuron loss (Xu et al., 2017), occurred at different time points after static chronic spinal cord compression. Nevertheless, dynamic neurophysiological and hemodynamic changes and their potential mechanisms at various neck positions remain largely unknown. In this study, we used data from a retrospective CSM cohort study and CSM rat models to investigate the neurophysiological changes and their correlations with hemodynamic responses at dynamic neck positions under CSM conditions, as well as the potential pathophysiological mechanisms behind.

## Materials and Methods

### Study Design

**FIGURE 1:**
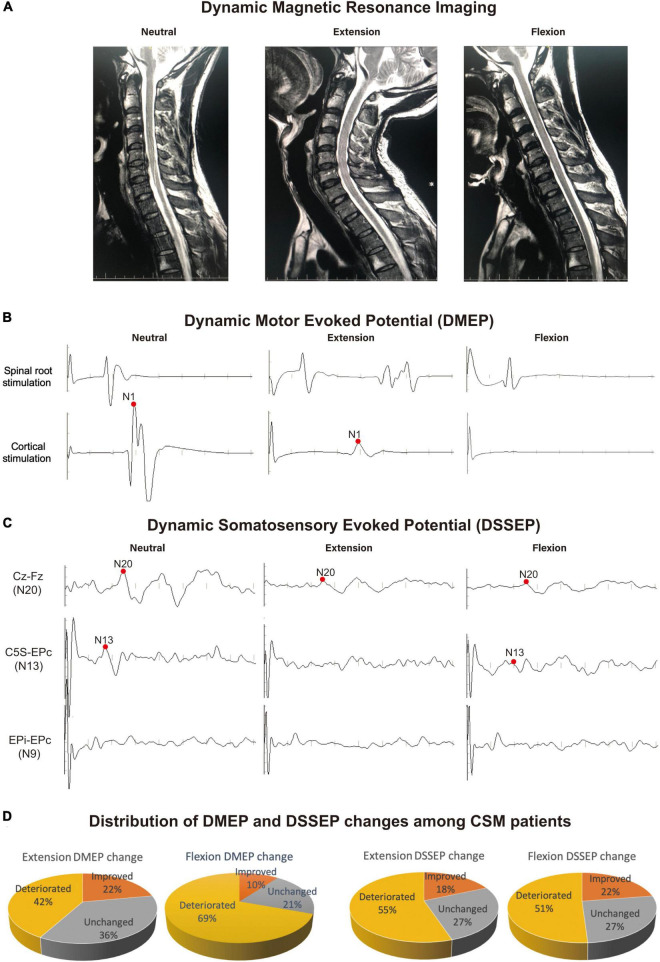
Example of the most frequent dynamic electrophysiological pattern encountered in CSM patients. Recordings were taken from a 39-year-old CSM patient complaining of leg fatigue, paresthesia of the feet and clumsy hands. **(A)** Dynamic magnetic resonance imaging (DMRI) disclosed a protruding disk and narrowing of the cervical canal at C5/6 level upon neutral position. The cord was compressed more severely from both the anterior and posterior directions at C5/6 level upon extension. Upon flexion, the cord was draped forward to the protruding disk on C5/6 segment. **(B,C)** The patient showed normal dynamic motor evoked potentials (DMEPs) and dynamic somatosensory evoked potentials (DSSEPs) at neutral position. Upon extension, the patient had disappeared DSSEP N13 wave, and compromised DMEP N1 and DSSEP N20 wave. Upon flexion, the patient had disappeared DMEP wave, and compromised DSSEP N13 and N20 waves. **(D)** Distribution of patients with different DMEP and DSSEP changes in the study sample. There were 42% and 69% patients had deteriorated DMEPs upon extension and flexion, respectively. CSM patients were significantly more likely to suffer DMEP deteriorations upon neck flexion than upon neck extension (Chi-square, *P* < 0.001), while there was no difference in the distribution of DSSEP-deteriorated patients upon extension and flexion. Data were generated from 49 CSM patients.

**FIGURE 2:**
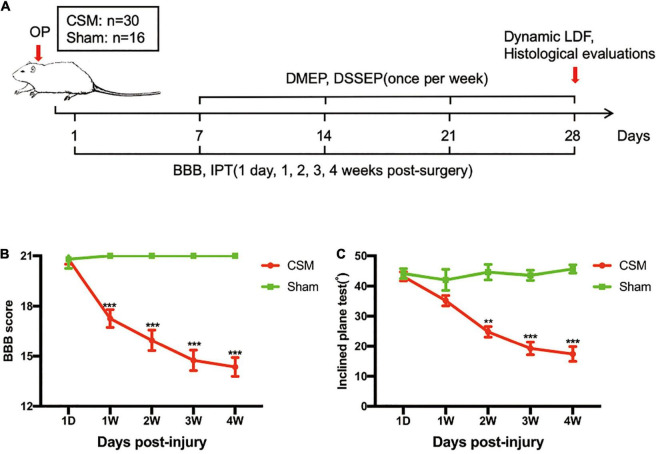
Experimental design and neurobehavioral tests of the CSM and sham-operated rats. **(A)** Experimental design. **(B)** Animals were tested weekly starting from 1 day post-injury (dpi) in an open field, and motor function recovery was evaluated according to the Basso, Beattie, and Bresnahan (BBB) score. **(C)** Animals were tested weekly starting from 1 dpi using the inclined plane test (IPT), and the angle (°) was recorded to reveal motor function recovery. **p < 0.01, ***p < 0.001.

All experimental procedures were approved by the Research Ethics Committee of Sun Yat-sen University, Guangzhou, China and conformed to all relevant regulatory standards. We used only recorded medical data of consecutive CSM patients at our department with preoperative DMEP and DSSEP examinations between 2015 and 2019 to investigate neurophysiological and functional outcomes in CSM patients (Figure 1). The study was retrospectively registered on April 30th, 2020 (Trial registration number: [2020]151) and 49 patients were finally enrolled in this study. A total of 46 male adult Sprague–Dawley (SD) rats (250–400 g) were randomly allocated to the sham (*n* = 16) and CSM (*n* = 30) groups. The animals in the CSM group underwent implantation of a water-absorbing polymer sheet into the cervical spinal canal, which expanded over time to induce chronic compression of the cord. Neurological functions were evaluated by behavioral tests from 1 day post-injury (dpi) to 4 weeks post-injury (wpi), and DMEPs and DSSEPs tests from 1 to 4 wpi. At 4 wpi, the structural changes of the cord at various neck positions were evaluated by dynamic MRI scans. The perfusion status of the cord at dynamic positions was assessed by LDF. The ultrastructure of the cord was evaluated by routine histology (Figure 2A).

### Electrophysiological Evaluations for Cervical Spondylotic Myelopathy Patients

An electrophysiological monitoring system (Nicolet Endeavor CR) was used to elicit and record the DMEP and DSSEPs. Compound muscle action potentials (CMAPs) to magnetic stimulation of motor cortex and roots (Magstim 200; large coil with outer diameter of 14 cm) were recorded according to recommendations of the *ad hoc* IFCN committee (Rossini et al., 1994). To evoke muscle responses with the shortest latencies and maximal amplitudes, cortical stimuli were applied while the subjects exerted a moderate isometric voluntary contraction of the target muscle abductor digiti minimi (ADM). Cervical motor roots were optimally stimulated when the coil was centered over spinous processes, its inner edge overlooking the foraminal exit of the roots tested. For MEP testing, CMAPs were recorded with adhesive surface electrodes with a belly-tendon montage. Signals were filtered (30 Hz–3 kHz), amplified with two different gains (1 mV/V and 100 mV/V) for accurate discrimination of amplitude and latency, and stored on hard disk for further analysis. At least five consecutive trials were collected and superimposed. Central motor conduction time (CMCT) was calculated by subtracting the latency of responses to spinal root stimulation from the minimal latency of responses to cortical stimulation.

Median and ulnar nerve DSSEPs were examined using established methods described in our previous study (Qi et al., 2019; Yu et al., 2020). We compared the same patient’s median nerve SSEP upon extension or flexion to those in the neutral position. Recording electrodes were placed over the spinous process of the 2nd cervical vertebra (C2S), the contralateral parietal cortex (Cc) and forehead reference site (Fz) regions of the scalp, and Erb’s points ipsilateral (EPi) and contralateral (EPc) to the stimulation (Nuwer et al., 1994). The DSSEP waves for each recording montage labeled EPi-EPc, C5S-EPc, and Cc-Fz were recorded as N9, N13, and N20, respectively. We have adopted N9 as the standard reference channel. When N9 was unidentifiable or poorly reproducible, the existence of peripheral nerve pathology is suspected.

A DMEP/DSSEP improvement (or deterioration) upon extension or flexion is defined as a shortened (or prolonged) MEP N1 or DSSEP N13 wave latency exceeding 2.5 SD of that at neutral position (which were 2.3 ms for MEP N1 wave and 1.78 ms for SSEP N13 wave in this study); or increased (or decreased) amplitude exceeding 50% compared with the patient’s MEP/SSEP in the neutral position. We define an immeasurable MEP/SSEP as a waveform that could not be identified by averaging over 500 sweeps. Any measurable MEP/SSEP waveform would be considered a MEP/DSSEP improvement compared to an immeasurable SSEP waveform.

### Induction of Cervical Spondylotic Myelopathy Rat Models

Each rat in the sham and CSM groups was anaesthetized with 10% chloral hydrate (300 mg/kg) (Guangzhou FISCLAB Environ. Sci-Tech. Co., Ltd., Guangzhou, China). Following exposure of the spinal process and laminae of C4–C6 from the posterior, the ligamentum flavum and C5 lamina were removed to access the epidural space. In the CSM group, the polymer (1→4)-3,6-anhydro-a-l-galactopyranosyl-(1→3)-β-D-galactopyranan) sheet (1 mm × 3 mm × 1 mm) was implanted into the C6 epidural space on the dorsal part of the spinal cord. Spinal cord compression was achieved by expanding the polymer caused by liquid absorption (Long et al., 2014; Cheng et al., 2015). This polymer sheet can absorb liquid in the spinal canal to expand its volume sevenfold (approximately 2.3 mm × 4.2 mm × 2.2 mm). In the sham group, the C5 lamina was removed without insertion of the polymer sheet. Following surgery, the incision was closed in layers with complete hemostasis. To prevent dehydration, animals received a subcutaneous (s.c.) injection of lactated Ringer’s solution (200 μL) immediately after surgery. All rats were administered an intramuscular injection of penicillin G (80 U/g) during surgery to prevent infection, and carprofen (4–5 mg/kg, Rimadyl, Pfizer) was injected subcutaneously 2 days post-surgery for further pain relief as needed. All surgeries were performed by the same experienced investigator.

### Neurobehavioral Assessments

To evaluate motor functional recovery, the Basso, Beattie, and Bresnahan (BBB) locomotor scale (Basso et al., 1995) and an IPT (Rivlin and Tator, 1977) were conducted at 1 dpi and 1, 2, 3 and 4 wpi. BBB scores ranged between 0 and 21; a score of 0 reflected complete paralysis and a score of 21 indicated normal locomotion. Lower scores (0–7) denoted isolated joint movements with little or no hindlimb movement; intermediate scores (8–13) indicated intervals of uncoordinated stepping; and higher scores (14–21) signified forelimb and hindlimb coordination. For the IPT, rats were placed horizontally on a smooth, tilted board. The board was initially placed in a horizontal orientation (0°), and the angle of the board was increased by 5°–10° after each attempt. The maximum angle at which the rats remained on the board for 10 seconds was recorded. The evaluation was conducted by two investigators blinded to the group assignments.

### Electrophysiological Evaluations for Cervical Spondylotic Myelopathy Rats

**FIGURE 3:**
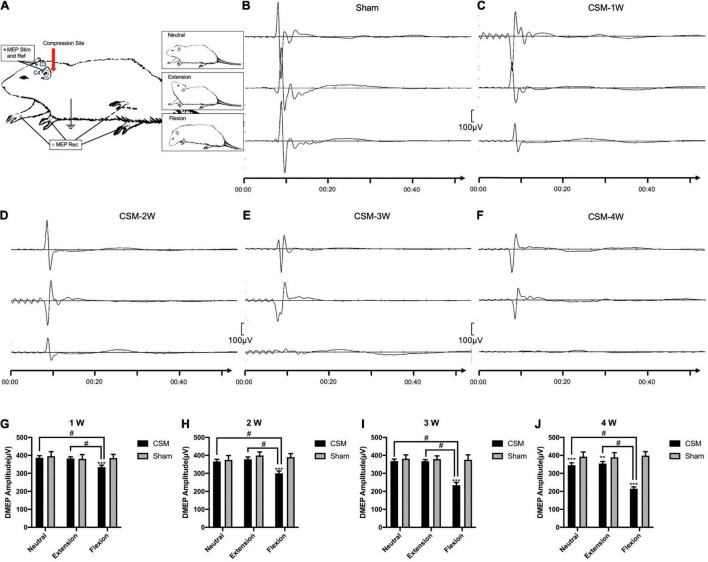
Dynamic motor evoked potentials (DMEPs) illustrating. **(A)** Electrode placement. To elicit the motor evoked potentials (MEPs), anodal stimuli (Stim) and a reference (Ref) electrodes were applied to the skull (C3 and C4). The responses were recorded from electrodes on the extensor digitorum communis in the forelimb and tibialis anterior in the hindlimb. **(B)** Representative images showing the N1 DMEPs in a sham group rat. The upper, middle and lower waves were DMEPs performed upon neutral, extension and flexion positions, respectively. **(C–F)** Representative images showing the N1 DMEPs in different time points of the CSM rats. The N1 waves of representative CSM rats at 3 and 4 weeks post-surgery upon flexion were abolished. **(G–J)** Quantification of DMEP amplitudes in CSM and sham groups at each time point. The DMEPs did not change at all neck positions after 10 min of implanting the unexpanded compression material. The DMEP amplitudes in CSM group did not change upon neutral and extension until 4 weeks post injury compared with the sham group. Upon flexion, the DMEPs in the CSM group deteriorated significantly compared with the other two neck positions, also with the flexion DMEPs in the sham group. **, *** indicated statistical significance (*p* < 0.01, 0.001, respectively) in the difference between the Sham and CSM group using *t*-test. # indicated statistical significance (*p* < 0.05) in the difference between different neck positions using one-way ANOVA and *post hoc* test.

**FIGURE 4:**
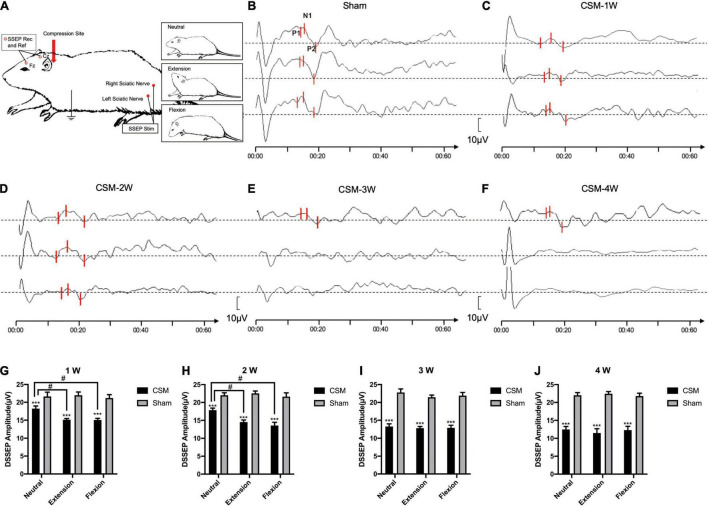
Dynamic somatosensory evoked potentials (DSSEPs) illustrating. **(A)** Electrode placement. To elicit the somatosensory evoked potentials (SSEPs), stimuli (Stim) were applied to the sciatic nerve and recorded from the Fz and Cz points on the skull. **(B)** Representative images showing the DSSEP waves in a sham group rat. The upper, middle, and lower waves were DSSEPs performed upon neutral, extension and flexion positions, respectively. **(C–F)** Representative images showing the DSSEPs in different time points of the CSM rats. P1, N1, P2 peaks on each wave were marked with three bars, respectively. The amplitude of each wave was defined as the peak-to-peak amplitude of the N1 and P2 waves. The waves of representative CSM rats at 3 and 4 weeks post-surgery upon extension and flexion were abolished. **(G–J)** Quantification of DSSEP amplitudes in CSM and sham groups at each time point. The latency and amplitude of DMEPs peaks in the sham group upon all three positions remained unchanged at all testing time points. From 1 to 4 weeks of chronic compression injury, increasing numbers of rats showed abolished SSEP with the increase of compression time. The CSM rats had more abolished DSSEP waves and significantly lower DSSEP amplitude than the sham models upon all neck positions at each time point after injury (*t*-test, *p* < 0.001). At the first 2 wpi, the CSM rats had less abolished waves and significantly higher (ANOVA *post hoc* test, *p* < 0.05) DSSEP amplitude upon neutral compared with the other two neck positions. The DSSEP amplitudes at all three neck positions were similar after 3 wpi. **, *** indicated statistical significance (*p* < 0.01, 0.001, respectively) in the difference between the Sham and CSM group using *t*-test. # indicated statistical significance (*p* < 0.05) in the difference between different neck positions using one-way ANOVA and *post hoc* test.

The functional integrity of the spinal cord among the model rats at dynamic positions was evaluated by DMEPs and DSSEPs at 1 to 4 wpi. An electrophysiological monitoring system (Nicolet Endeavor CR) was used to elicit and record transcranial electrical stimulation motor evoked potentials (TES-MEPs) and SSEPs. The animals were evaluated under general anesthesia with 10% chloral hydrate (300 mg/kg) (Guangzhou FISCLAB Environ. Sci-Tech. Co., Ltd., Guangzhou, China) intraperitoneally. Their scalps and the posterior portion of their necks were shaved and aseptically treated using iodine. Two electrodes were placed 2 mm posterior to bregma and 3 mm to the left and right of the longitudinal midline; these locations correspond to the left or right primary motor cortex (C3 and C4), respectively, and served as DMEP stimulation electrodes and a reference electrode alternatively. Another two electrodes were placed at the midline of the skull 2 mm anterior to bregma (Fz) and at the midpoint of the ears (Cz) for DSSEP recording. Two electrodes were placed around each side of the sciatic nerve along the course of the biceps femoris muscle for DSSEP stimulation. Two were placed on each side of the extensor digitorum communis in the forelimb and tibialis anterior in the hindlimb for DMEP recording. A ground electrode was placed on the back of the subject subcutaneously. Rats were positioned neutrally at approximately 40° extension and then at approximately 40° flexion of the cervical spine using an angle-adjustable stereotaxic frame for dynamic MEP and SSEP measurements (Figures 3A, 4A).

Constant current stimulation at the skull was used for the generation of MEPs. Single-trial MEPs were obtained with a current intensity of up to 16 mA and a pulse width of 50 ls at a frequency of 350 Hz for a 1-min duration. MEPs were recorded using subdermal needle electrode pairs from the extensor digitorum communis in the forelimb and tibialis anterior in the hindlimb.

Constant current stimulation around sciatic nerves with a magnitude of 6 mA, duration of 0.02 ms, and frequency of 3.43 Hz was used to elicit DSSEPs. Cortical SSEPs were recorded from the skull at Cz–Fz. We averaged 256 SSEP trials to improve the signal-to-noise ratio. SSEP signals were filtered using a bandpass filter of 10 Hz to 250 Hz. A sensitivity of 20 lV/div and a time base of 5 ms/div were used to display the SSEP responses.

Onset latency and peak-to-peak amplitude of the responses at dynamic positions were measured at the endpoint of the study. The onset latency was measured from the delivery of the stimulus to the first positive or negative deflection (N1 in Figure 4) from baseline. Peak-to-peak amplitude is defined as the maximum amplitude between the largest positive and negative peak (N1-P2 in Figure 4). Each MEP/SSEP test was repeated three times, and their average value was taken. We define an immeasurable MEP/SSEP as a waveform that could not be identified by averaging over 500 sweeps. SSEP and MEP responses recorded from each limb were classified separately. For the final analysis, the lowest MEP or SSEP amplitude among the four limbs was utilized as the definitive data point.

### Dynamic Magnetic Resonance Imaging Evaluations

Cord compression was evaluated by dynamic MRI using a 3.0-T MR imager (Siemens Trio). The animals were anaesthetized by isoflurane inhalation, lied in prone, 40° extension and 40° flexion positions, with a surface coil placed over the animals’ cervical spine region to acquire anatomical T2WIs. T2WIs were acquired with the following parameters: echo time (Puente et al., 2015)/repetition time (TR) = 35/2500 ms (T2W) and 115/2500 ms (PDW), slice thickness = 1 mm, interslice distance = 1.1 mm, and number of excitations (NEX) = 4. A total of 15 axial slices covering C3–C7 of the cervical spinal cord were acquired at each disk and body level. The image slice planning was the same as that in anatomical axial images, with 15 slices covering the cervical spinal cord from C3 to C7.

The sagittal diameters of the spinal canals and spinal cords and the transverse diameters of the cords and canals were measured with Osirx (Pixmeo, Geneva, Switzerland), which is a standard software for MR equipment packages. MR signal intensity was analyzed in regions of interest (ROIs) at the compression site (or C6 levels in the sham group) of the spinal cord with Osirx. We used the T2WI intensity signals of paraspinal muscles as calibration references in each MR image and quantified the relative signal intensities of the ROI in cervical gray matter in both sham and CSM rats. The relative signal intensity was calculated by dividing the signal intensity of the ROI in cervical gray matter by that of paraspinal muscles.

### Dynamic Laser Doppler Flowmetry Measurements

We measured the SCBF and oxygen saturation (SO_2_) of the rats at 10 min and 4 weeks after implanting the compression material in the CSM group or exposing the dorsal dura mater in the sham group. For SCBF and SO_2_ measurements, an OxyFlo (ADInstruments Pty Ltd., Castle Hill, Australia) LDF probe was attached to the stereotaxic frame and positioned in contact with the dorsal dura mater at the right side of the central vein at the C3 and C6 segmental levels of the spinal cord. Upon neutral positioning, after adjusting the SCBF at C3 to the same position (580 PU) for both the sham and CSM rats, we monitored the SCBF at the C6 (compression) level in the sham and CSM rats. The rats were then positioned upon maximum cervical extension and cervical flexion postures for at least 5 min before undergoing the same recording process. Each recording process started with an adjustment of the SCBF data at the C3 level, and the recording time at C6 or the injury level lasted for at least 1 min. Output signals were recorded in Powerlab (ADInstruments Pty Ltd., Castle Hill, Australia) continuously throughout the experiment and averaged every 3 s.

### Histological and Immunohistochemistry Evaluations

In brief, rats in different groups were euthanized with an overdose of intravenous sodium pentobarbital and transcardially perfused with 0.9% saline followed by 4% paraformaldehyde in 0.1 M phosphate buffer (PFA) at the endpoint of the study. The C5–C7 spinal cord was harvested, fixed overnight with 4% formaldehyde in phosphate-buffered solution at 4°C, and embedded in paraffin. A series of 25-μm thick spinal cord sections were used for HE and immunohistochemical (IHC) staining. Microwave epitope retrieval was performed before staining. Sections were incubated overnight at 4°C with rabbit anti-NeuN (1:150; Abcam, Cambridge, United Kingdom), rabbit anti-CD31 (1:200; Abcam, Cambridge, United Kingdom), rabbit anti-von Willebrand factor (vWF) (1:200; Abcam, Cambridge, United Kingdom), or mouse anti-vascular endothelial growth factor (VEGF) (1:200; Abcam, Cambridge, United Kingdom) antibodies. Subsequently, the sections were incubated with a ready-to-use DAKO ChemMate EnVision™ kit (K500711; Dako; Agilent Technologies, Inc., Santa Clara, CA, United States) for 30 min at room temperature. Images of each section of the perilesional spinal cord were acquired at 4×, 10×, or 20× magnification using an XC30 camera mounted on an Olympus microscope (Olympus Corporation, Tokyo, Japan).

Motor neurons have large nuclei and well-developed, densely stained Nissl bodies in the cytoplasm. Since NeuN protein appears in neuron-specific nuclei, the nucleus of the motor neuron is more clearly detected with the characteristic large nucleolus [uniform diameter of approximately 5 μm-diameter (Molander et al., 1989)] in NeuN staining compared with H-E staining. Those large-nuclear NeuN+ cells in the anterior horns were regarded as motor neurons and counted manually in five random 20× magnification visual fields in both sides of the spinal cords (counting frame area, 240 × 320 μm^2^) from 10 consecutive slices of five rats from each group. We also counted the NeuN+ cells in the dorsal horns (not specified as motor neurons) of these rats in the same method. In each visual field, large-nuclear NeuN+ motor neurons in the anterior and all NeuN+ cells in the dorsal horns were manually counted using a handheld tally counter for three times. The obtained cell counts were averaged to give a representative count for each specific region.

CD31, vWF, and VEGF immunodensities were quantified in five random 20× magnification visual fields (quantification area, 240 × 320 μm^2^) in both sides of the anterior and posterior horns using ImageJ software (US National Institutes of Health, Bethesda, MD, United States) from 10 consecutive slices of each rat. Images were converted to grayscale and subjected to threshold evaluations. CD31, vWF, and VEGF were quantified as the fraction of the area above the threshold within the region of interest, and the relative optical densities (ROD) were calculated. Each visual field was taken and analyzed for three times. The obtained RODs were averaged to give a representative value for each specific region.

### Statistical Analysis

Comparisons of the ultrastructure of the spinal cord on the injured and non-injured sides were performed using paired *t*-tests. Comparisons of the neural conduction situations of spinal cords among the animals categorized by DMEP and DSSEP responses and spinal cord perfusion status demonstrated by LDF at different neck positions were performed using one-way analysis of variance (ANOVA) and *post hoc* tests. The level of significance was set at *p* < 0.05. All data analyses were performed using SPSS 15.0 analysis software (SPSS Inc., Chicago, IL, United States).

## Results

### Dynamic Neurological Dysfunction in Cervical Spondylotic Myelopathy Patients

Neurological outcomes were evaluated using data from 49 CSM patients enrolled in a retrospective cohort study (Table 1). In this population, we found compression of the spinal cord most commonly occurred at C4/5 and C5/6 (Figure 1A shows a patient with a C5/6 disk protrusion). The DMEP amplitude ratio upon flexion was significantly smaller than that upon extension (*t*-test, *P* = 0.032), while the DSSEP amplitude ratio did not vary significantly upon extension and flexion (Figures 1B,C). We also classified patients into groups based on their DMEPs or DSSEPs changes. Twenty and 34 patients had deteriorated DMEPs upon extension and flexion, respectively, while 27 and 25 had deteriorated DSSEPs upon extension and flexion, respectively (Figure 1D). CSM patients were significantly more likely to suffer DMEP deteriorations upon neck flexion than upon neck extension (Chi-square, *P* < 0.001), while there was no difference in the distribution of DSSEP-deteriorated patients upon extension and flexion (Chi-square, *P* = 0.568).

**TABLE 1:** General characteristics and dynamic neurophysiological changes for 49 patients enrolled in the CSM cohort study.

Variable	Descriptive statistics
Gender (female/male)	21/28
Age	55.8 ± 11.3
Stenotic levels (C2/3, C3/4, C4/5, C5/6, C6/7, C7/T1)	2, 22, 37, 37, 21, 3
Baseline severity score (mJOA)	14.84 ± 1.78
Duration of symptoms (months)	26.82 ± 27.91
DMEP amplitude ratio upon extension (%)	81 ± 37
DMEP amplitude ratio upon flexion (%)	67 ± 21*
DSSEP amplitude ratio upon extension (%)	72 ± 33
DSSEP amplitude ratio upon flexion (%)	74 ± 32
Number of patients with DMEP change upon extension (improved/unchanged/deteriorated)	11/18/20
Number of patients with DMEP change upon flexion (improved/unchanged/deteriorated)	5/10/34^#^
Number of patients with DSSEP change upon extension (improved/unchanged/deteriorated)	9/13/27
Number of patients with DSSEP change upon flexion (improved/unchanged/deteriorated)	11/13/25

*Indicated statistical significance (p < 0.05) in the difference between the DMEP amplitude ratio upon neck extension and flexion positions using unpaired t-test.

*^#^Indicated statistical significance (p < 0.001) in the difference between the number of patients with or without DMEP deterioration upon neck extension and flexion positions using Chi-square test.*

### Neurological Dysfunction in Cervical Spondylotic Myelopathy Rats

To parallel the clinical data, neurobehavioral analyses were performed in a CSM rat model in a blinded, randomized experiment (Figure 2A). Following incomplete spinal cord compression, progressive neurological deteriorations have been observed in animal models, as previous studies have indicated (Dhillon et al., 2016). The BBB scores and IPT angles of the CSM rats gradually decreased from 1 dpi to 4 wpi, and were significantly lower than those of the sham models after one and two wpi (Figures 2B,C).

Dynamic motor evoked potentials were tested at 1, 2, 3, and 4 wpi, following the protocol described in the Methods section (Figure 3A). The number of rats with abolished waves and the latency and amplitude data of the remaining rats are summarized in Table 2. The DMEP results of the sham group remained unchanged among all neck positions and testing time points (Figure 3B). From 1 to 3 wpi, the DMEP amplitudes of the CSM rats were slightly affected upon neutrality and extension but were significantly diminished upon flexion (*t*-test, *p* < 0.001) compared with that of the sham models (Figures 3C–E,G–I). At 4 wpi, the DMEP latencies and amplitudes of the CSM rats were significantly diminished at all neck positions compared with the sham rats (Figures 3F,J), indicating permanent motor transduction deficit regardless of the neck postures. It should be noted that the CSM rats had the worst DMEP latencies and amplitudes (ANOVA *post hoc* test, *p* < 0.001) and the most abolished DMEP waves (Chi-square, *p* < 0.05) upon flexion compared with the other two positions at any time point after the compression injury (Figures 3G–J).

**TABLE 2:** DMEPs in Sham and CSM rats after compression injury.

	Sham			CSM		
	Neutral	Extension	Flexion	Neutral	Extension	Flexion
**1 Wpi**						
Latency (ms)	7.96 ± 1.55	8.08 ± 1.07	8.54 ± 1.26	8.31 ± 1.16	8.52 ± 1.54	10.2 ± 1.41^#^*
Amplitude (μV)	395.67 ± 45.61	380.48 ± 43.59	385.27 ± 36.78	386.98 ± 28.38	382.56 ± 23.84	334.19 ± 26.56^#^***
Immeasurable waves (*n*)	0	0	0	1	1	4
**2 Wpi**						
Latency (ms)	7.98 ± 1.24	7.99 ± 1.24	7.73 ± 1.31	8.33 ± 1.55	8.75 ± 1.86	10.98 ± 1.82^#^*
Amplitude (μV)	374.84 ± 43.74	399.4 ± 35.67	390.12 ± 34.88	366.42 ± 29.74	378.58 ± 32.19	300.02 ± 30.56^#^***
Immeasurable waves (*n*)	0	0	0	1	1	6^#^
**3 Wpi**						
Latency (ms)	7.74 ± 1.21	8.01 ± 1.39	7.79 ± 1.24	9.42 ± 2.18*	9.67 ± 2.29*	11.68 ± 2.65^#^*
Amplitude (μV)	381.25 ± 38.86	392.4 ± 43.88	375.78 ± 49.74	368.21 ± 29.23	367.77 ± 22.35	234.75 ± 31.12^#^***
Immeasurable waves (*n*)	0	0	0	2	2	10^#^
**4 Wpi**						
Latency (ms)	7.83 ± 1.34	7.52 ± 1.19	7.95 ± 1.42	9.61 ± 2.33*	9.42 ± 1.84*	12.57 ± 2.68^#^*
Amplitude (μV)	392.66 ± 47.71	389.42 ± 46.56	398.58 ± 39.94	345.3 ± 34.43***	353.85 ± 31.19**	215.39 ± 18.82^#^***
Immeasurable waves (*n*)	0	0	0	4	4	13^#^

*, **, *** Indicated statistical significance (p < 0.05, 0.01, 0.001, respectively) in the difference between the Sham and CSM group using t-test.

*^#^Indicated statistical significance (p < 0.05) in the difference between various neck positions using one-way ANOVA and post hoc test.*

Dynamic somatosensory evoked potentials were assessed for each rat following the DMEPs test (Figure 4). The detailed data are summarized in Table 3. The DSSEP results of the sham group remained unchanged among all neck positions and testing time points (Figure 4B). Increasing numbers of rats showed abolished SSEPs with the increasing compression time from 1 to 4 wpi. At the first 2 wpi, CSM rats had fewer abolished waves and significantly higher (ANOVA *post hoc* test, *p* < 0.05) DSSEP amplitudes at neutral positions than at the other two neck positions (Figures 4C,D,G,H). However, the DSSEP amplitudes upon all three neck positions were similar at 3 and 4 wpi (Figures 4E,F,I,J), indicating permanent somatosensory transduction deficit regardless of the neck postures. The CSM rats had always a larger number of abolished DSSEP waves and significantly lower DSSEP amplitudes than the sham rats at all neck positions after the compression injury (*t*-test, *p* < 0.001).

**TABLE 3:** DSSEPs in Sham and CSM rats after compression injury.

	ShamM			CSM		
	Neutral	Extension	Flexion	Neutral	Extension	Flexion
**1 Wpi**						
Latency (ms)	15.24 ± 2.81	15.97 ± 3.47	16.11 ± 3.55	16.43 ± 3.22	17.19 ± 3.06	16.33 ± 2.58
Amplitude (μV)	21.66 ± 2.15	21.99 ± 1.63	21.23 ± 1.7	18.25 ± 1.85***	15.13 ± 0.79^#^***	15.08 ± 1.09^#^***
Immeasurable waves (*n*)	0	0	0	2	7	3
**2 Wpi**						
Latency (ms)	15.68 ± 2.32	15.94 ± 3.28	16.42 ± 3.17	15.94 ± 3.02	18.26 ± 3.41	17.33 ± 2.95
Amplitude (μV)	22.03 ± 1.25	22.54 ± 1.17	21.63 ± 1.94	17.87 ± 1.32***	14.57 ± 1.07^#^***	13.59 ± 2.06^#^***
Immeasurable waves (*n*)	0	0	0	4	11	7
**3 Wpi**						
Latency (ms)	16.24 ± 3.67	15.77 ± 2.56	15.99 ± 2.89	17.71 ± 4.37	17.93 ± 4.79	16.21 ± 6.72
Amplitude (μV)	22.8 ± 1.8	21.46 ± 1.09	21.9 ± 1.66	13.3 ± 1.52***	12.84 ± 0.86***	12.92 ± 1.36***
Immeasurable waves (*n*)	0	0	0	11	13	14
**4 Wpi**						
Latency (ms)	15.99 ± 3.02	15.64 ± 2.71	16.05 ± 2.77	17.56 ± 4.83	18.32 ± 5.4	16.3 ± 5.11
Amplitude (μV)	22.03 ± 1.32	22.44 ± 1.12	21.8 ± 1.43	12.49 ± 1.4***	11.51 ± 1.88***	12.32 ± 1.37***
Immeasurable waves (*n*)	0	0	0	16	17	20

*** Indicated statistical significance (p < 0.001) in the difference between the same neck position in Sham and CSM group using t-test.

*^#^Indicated statistical significance (p < 0.05) in the difference between various neck positions using one-way ANOVA and post hoc test.*

### Dynamic Cervical Cord Compression in Cervical Spondylotic Myelopathy Rats

**FIGURE 5:**
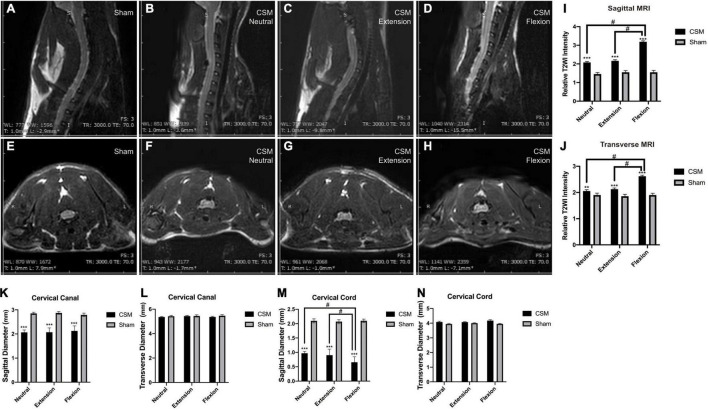
The MRI signal intensity changes in different neck positions of CSM rats. **(A,E)** Representative images showing neutral position of a rat in the sham group. There was no material compressing the spinal cord. **(B,F)** Representative images showing neutral position of a CSM rat model. The compression material is localized on the posterior side at the C5/6 level of the cord. There existed a significantly higher T2WI contrast medium intensity at the injured site upon neutral position of a CSM rat compared with a sham model. **(C,G)** Representative images showing extension position of a CSM rat model. **(D,H)** Representative images showing flexion position of a CSM rat model. We found a significant hyperintense “pencil-like” lesion pattern on sagittal T2WIs and the so-called “snake eyes” with hyperintense signal conversion on axial T2WIs of CSM rats upon flexion. **(I,J)** Upon flexion, the T2WI signals of the cord at both sagittal and transverse sections were significantly more intense than that upon neutral or extension positions in CSM rats. **(K,M)** Histograms show the sagittal diameters of the spinal canal and spinal cord in the CSM group were significantly smaller than that in the sham group at all neck positions. At dynamic neck positions, the sagittal diameter of the cervical cord was the smallest upon flexion in CSM models, while remained unchanged in the sham group. **(L,N)** The transverse diameters of the canal and cord were similar between the CSM and sham groups and did not change at dynamic neck positions. **, *** indicated statistical significance (*p* < 0.01, 0.001, respectively) in the difference between the Sham and CSM group using *t*-test. # indicated statistical significance (*p* < 0.05) in the difference between different neck positions using one-way ANOVA and *post hoc* test.

To explore the compression degrees and inner structural changes in CSM rats at dynamic neck positions, we performed dynamic MRI on the cervical cord region of our models at 4 wpi. Compared with the sham model, compression was clearly evident at the posterior side of the spinal cord at the C6 region of CSM rats (Figures 5A–H). The sagittal diameters of the spinal canal (1.89 ± 0.11 mm) and spinal cord (0.97 ± 0.1 mm) in the CSM group were significantly smaller than that (canal: 2.85 ± 0.09 mm, cord: 2.10 ± 0.04 mm) in the sham group (*t*-test, *p* < 0.001) at all neck positions (Figures 5K,M). At dynamic neck positions, the sagittal diameter of the cervical cord was the smallest upon flexion in CSM models (*t*-test, *p* < 0.05), while remained unchanged in the sham group (Figure 5M). The transverse diameters of the canal (pooled mean 5.42 ± 0.06 mm) and cord (pooled mean 4.09 ± 0.04 mm) were similar between the CSM and sham groups, and did not change at dynamic neck positions (Figures 5L,N).

### Cervical Spondylotic Myelopathy Rats Showed Increased Intramedullary T2-Weighted Image Signal Intensity, Especially Upon Flexion

We then analyzed the relative T2WI signal intensities of the ROI in cervical gray matter in both sham and CSM rats. There was a significantly higher T2WI contrast medium intensity at the injured site in the neutral position in CSM rats (sagittal: 2.07 ± 0.16, transverse: 2.05 ± 0.19) than in sham rats (sagittal: 1.45 ± 0.14, transverse: 1.89 ± 0.13) (*t*-test, sagittal: *p* < 0.001, transverse: *p* = 0.002). T2WI intensity was even greater at the injured site of CSM rats in the flexion position (sagittal: 3.17 ± 0.17; transverse: 2.61 ± 0.15) than in the neutral (sagittal: 2.07 ± 0.16, transverse: 2.05 ± 0.19) or extension positions (sagittal: 2.15 ± 0.15, transverse: 2.12 ± 0.13) (*t*-test, *p* < 0.001) (Figures 5I,J).

### Cervical Spondylotic Myelopathy Rats Showed Decreased Spinal Cord Blood Perfusion, Especially Upon Flexion

**FIGURE 6:**
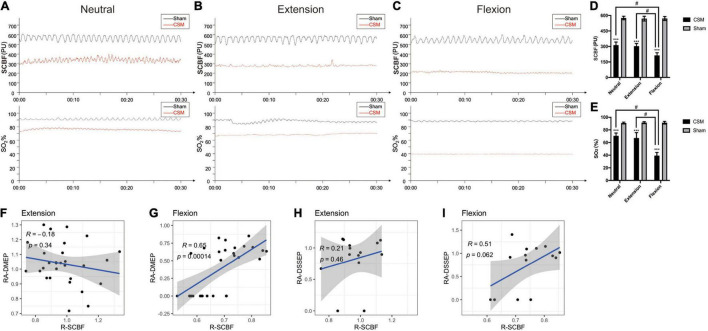
The alteration of instant spinal cord blood flow (SCBF) and oxygen saturation (SO_2_) level in different positions at 4-week post-injury, and its correlations with electrophysiological changes. **(A–C)** The maps of instant blood flow and oxygen saturation level were evaluated by a laser Doppler flowmetry at the C5/6 segment of the cervical cords upon neutral, extension and flexion positions in Sham and CSM groups. **(D,E)** Spinal cord blood flow (SCBF) and oxygen saturations (SO_2_%) at the compression sites of the CSM rats were significantly lower than that of sham models at all three neck positions (*p* < 0.001). Both of them also deteriorated significantly upon flexion (*p* < 0.001). **(F–I)** Pearson correlations between the relative change of SCBF (R-SCBF) and the relative amplitude of DMEPs (RA-DMEP) or DSSEPs (RA-DSSEP) upon extension and flexion. R-SCBF was significantly correlated with RA-DMEP upon flexion (Pearson correlation, *R* = 0.65, *p* < 0.001). *** indicated statistical significance (*p* < 0.001) in the difference between the Sham and CSM group using *t*-test. # indicated statistical significance (*p* < 0.05) in the difference between different neck positions using one-way ANOVA and *post hoc* test.

To determine whether the dynamic intramedullary T2WI hyperintensities were associated with the spinal cord perfusion change at dynamic neck positions, we performed dynamic LDF examinations to quantify the blood perfusion at the injury site of the spinal cord upon different neck postures. At 10 min after compression injury, the SCBF and SO_2_ were similar between the sham and CSM rats, indicating that there were hardly any effects of the unexpanded material on the perfusion of the cord at all neck positions (Supplementary Figure 1). At 4 wpi, the SCBF (pooled mean 572.69 ± 19.29 PU) and SO_2_ (pooled mean 91.29 ± 1.71%) in sham models remained unchanged in all three neck positions. For the CSM rats, their SCBF and SO_2_ at all three neck positions (neutral: SCBF = 382.6 ± 29.81 PU, SO_2_ = 70.63 ± 4.37%; extension: SCBF = 372.83 ± 24.81 PU, SO2 = 67.13 ± 8.43%; flexion: SCBF = 213.47 ± 25.55 PU, SO2 = 39.27 ± 4.97%) decreased significantly (*t*-test, *p* < 0.001) compared with the sham models. Furthermore, while the SCBF and SO_2_ did not change significantly from neutral to extension positions, both of these measures decreased significantly upon flexion in the CSM rats (*t*-test, *p* < 0.001) (Figures 6A–E). We also calculated the relative change in SCBF (R-SCBF) and oxygen saturation (R-SO_2_) upon extension or flexion by dividing each model’s SCBF and SO_2_% at extension or flexion by that at the neutral position. The R-SCBF at 4 wpi of CSM rats were 0.98 ± 0.14 and 0.73 ± 0.11 upon extension and flexion, respectively. The R-SO_2_ at 4 wpi of CSM rats were 0.96 ± 0.15 and 0.56 ± 0.09 upon extension and flexion, respectively. Both the R-SCBF and R-SO_2_ at flexion were significantly lower than that at extension (*t*-test, *p* < 0.001) for the CSM model at 4 wpi (Supplementary Figure 2).

### Correlations Between Dynamic Neurophysiological and Blood Flow Changes of the Spinal Cord

We calculated the relative amplitudes of DMEPs (RA-DMEP) and DSSEPs (RA-DSSEP) upon extension or flexion by dividing each model’s DMEP and DSSEP amplitude upon extension or flexion by that at neutral position. The immeasurable waveforms were also included with their amplitudes defined as 0 μV. The RA-DMEPs gradually decreased upon flexion and were significantly lower than those upon extension at each time point after injury (*t*-test, *p* < 0.05), while the RA-DSSEPs did not vary significantly upon extension and flexion (Supplementary Figure 3). Correlation analysis found that the R-SCBF was significantly correlated with the RA-DMEP (Pearson correlation, *R* = 0.65, *p* < 0.001) upon flexion, while there was no correlation between them upon extension. Neither the correlations between R-SCBF and RA-DSSEP at extension and flexion were significant (Figures 6F–I).

### Structural Damage and Neuronal Loss in Cervical Spondylotic Myelopathy Spinal Cords

**FIGURE 7:**
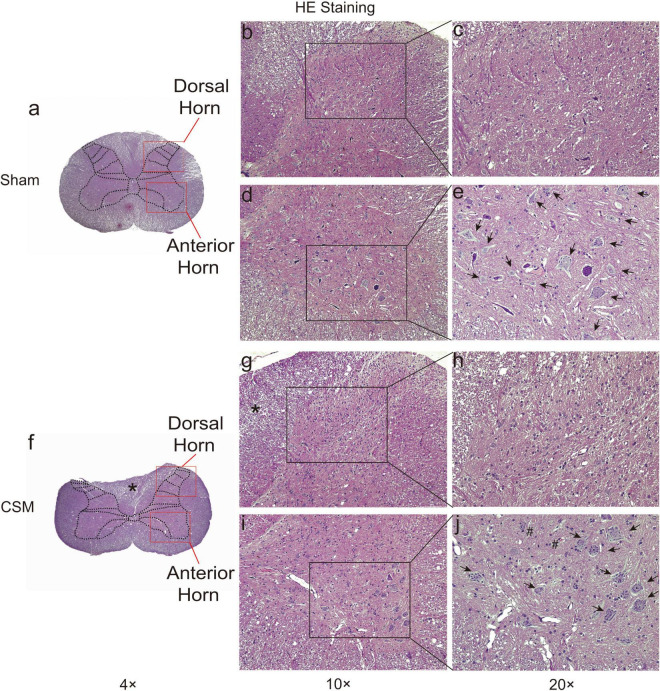
HE staining of cervical cords from Sham and CSM rats at 4-week post-injury. Representative HE staining of cervical spinal cord sections in Sham **(a–e)** and CSM **(f–j)** groups. **(b,c)** The gross and ultrastructure of the dorsal horn was intact in the sham group. **(d,e)** The gross and ultrastructure of the anterior horn was intact in the sham group. Motor neurons have large nuclei and well-developed, densely stained Nissl bodies in the cytoplasm (black arrows). **(f–h)** In a CSM model, the dorsal horn exhibit anatomical breakage. The dorsal funiculi suffered more severe breakage, with more significantly compressed and altered anatomical structures (*). **(i,j)** In a CSM model, the anterior horn of the gray matter also exhibited structural damage, including fragmentation of neuronal nuclei, pyknosis, neuropil damage, degradation of the extracellular matrix, cytoplasmic reduction, and cavity formation. Intratissue bleeding in the gray matter indicating disruptive circulation can also be observed (#) (magnification **a,f**: 4×; **b,d,g,i**: 10×; **c,e,h,j**: 20×).

**FIGURE 8:**
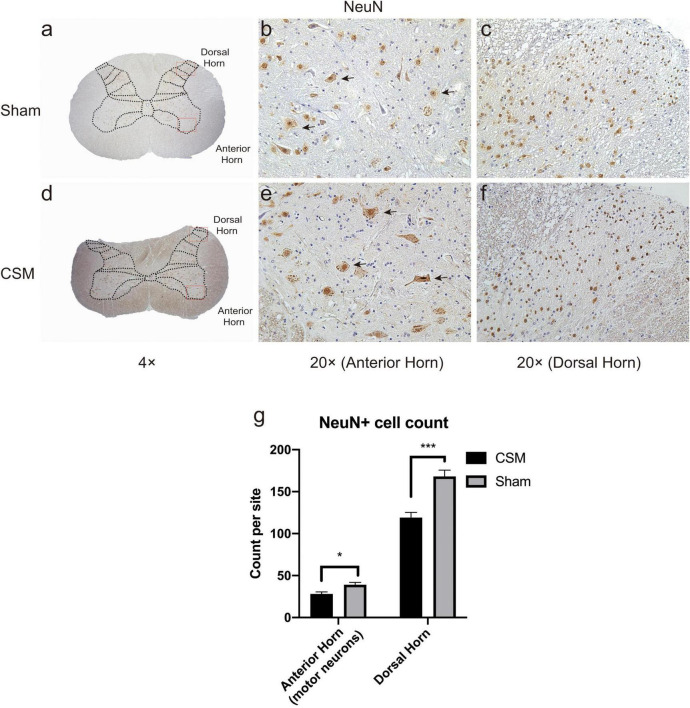
Chronic compression causes neuronal density decreases in both anterior and dorsal horns. Representative NeuN immunohistochemistry staining of transverse cervical spinal cord sections in Shan **(a–c)** and CSM **(d–f)** groups. **(a,d)** Gross view of cervical cords from the Sham and CSM models with a 4× magnification. **(b,e)** The large-nuclear NeuN+ cells in the anterior horns of Sham and CSM rats were regarded as motor neurons (black arrows indicated a few representative motor neurons) in 20× magnification visual fields. **(c,f)** NeuN+ neurons in the dorsal horns of Sham and CSM rats in 20× magnification visual fields. **(g)** The neuronal densities of both the anterior and dorsal horns of the gray matter in the CSM group were significantly lower than that in the sham group.

Hematoxylin and eosin staining revealed histological changes at the injury epicenter under different levels of magnification (Figure 7). The spinal cord was intact in the sham group. The polygonal Nissl bodies inside gray matter anterior horn neurons were large and dense. In a transverse section of a CSM model, the compressed spinal cord exhibited structural damage, including fragmentation of neuronal nuclei, pyknosis, neuropil damage, degradation of the extracellular matrix, cytoplasmic reduction, and cavity formation (Figures 7f–j). The dorsal funiculi suffered the most severe breakage (Figures 7g,h). We also observed intratissue bleeding in the gray matter, indicating disruptive circulation. Staining for NeuN in the anterior and dorsal horns of the gray matter was used to identify neurons and to estimate their number (Figures 8a–f). We found that the CSM rats had significant neuronal decreases in both the anterior (*t*-test, *P* < 0.05) and dorsal horns (*t*-test, *P* < 0.001) of the gray matter compared with the sham-operated rats at 4 wpi (Figure 8g). Structural damage in the cervical cords corresponded with neurobehavioral and neurophysiological deficits in CSM rats.

### Vascular Redistribution Underpins the More Severe Cervical Cord Ischemia Upon Flexion

**FIGURE 9:**
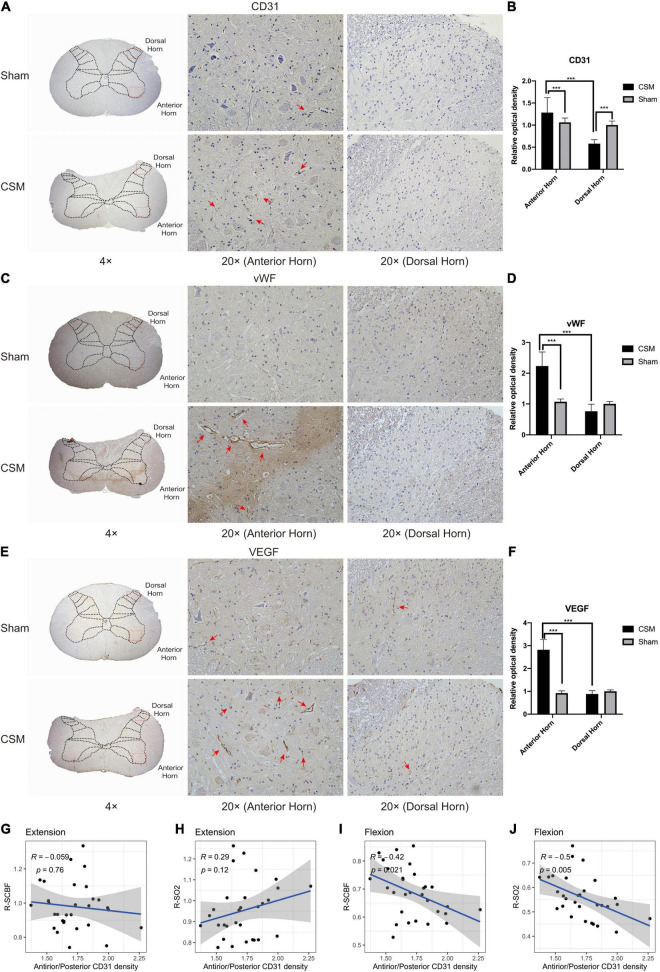
Chronic compression causes vascular redistribution and active angiogenesis in the anterior horn at 4-week post-injury. **(A)** Representative CD31 immunohistochemistry (IHC) staining of rat cervical spinal cord sections (4× and 20× magnification). Positive IHC staining of each marker was shown as brown part in each figure. The CD31 is a classical marker for assessing vascular density. Well-formed capillaries were mainly observed in the anterior horns (red arrows). **(C,E)** Representative vWF and VEGF immunohistochemistry staining of rat cervical spinal cord sections (4× and 20× magnification). The vWF and VEGF are classical markers for angiogenesis, which was more active in the anterior horns of the CSM rats (red arrows). **(B,D,F)** Expression levels of CD31, vWF, and VEGF were assessed by the relative optical density in IHC sections (20× magnification). **(G–J)** Pearson correlation analysis demonstrating that the relative CD31 expression between the anterior and posterior horns was not correlated with the relative change of spinal cord blood flow (R-SCBF) and spinal cord oxygen saturation (R-SO2) upon extension, but was significantly negatively correlated with both of them upon flexion (Pearson correlation, *p* < 0.05).

We found that the vascular densities assessed by CD31 immunochemical intensities (Bianconi et al., 2020) were significantly higher in the anterior horn and lower in the dorsal horn in CSM rats compared with those in the sham rats (*t*-test, *P* < 0.001) (Figures 9A,B). To determine the angiogenic activity, we performed IHC with angiogenic markers including vWF and VEGF (Starke et al., 2011). Angiogenesis in the gray matter anterior horns of CSM rats was significantly more active than that in their own dorsal horns or in both the anterior and posterior horns of sham-operated rats (*t*-test, *P* < 0.001) (Figures 9C–F). To examine whether uneven capillary distribution was associated with dynamic perfusion change at different neck positions, we calculated the Pearson correlations between the relative ratio of vascular densities as marked by CD31 at the anterior and posterior horns of the CSM rats’ gray matter and their dynamic LDF data (Figures 9G–J). The relative ratio of vascular densities between the anterior and posterior horn was negatively correlated with the R-SCBF (Pearson correlation, *R* = −0.42, *p* < 0.05) and R-SO2 (Pearson correlation, *R* = −0.5, *p* < 0.01) upon flexion, but was not significantly correlated with either of them upon extension.

Altogether, these data demonstrated chronic compression-induced vascular redistribution in the anterior and posterior parts of spinal cord parenchyma underpins the different SCBF change and subsequent neurophysiological alterations at dynamic neck positions.

## Discussion

Here, human clinical and electrophysiological data as well as rodent model experiments were used to delineate the mechanisms underlying the neurophysiological and hemodynamic responses at dynamic neck positions in the CSM conditions. Initially, sub-analysis of the retrospective CSM trial indicated dynamic neurological complications as well as more significant DMEP deterioration upon neck flexion in CSM. Rodent experiments demonstrated that flexion positioning increases the intramedullary T2WI signal intensity and exacerbates the ischemia of the spinal cord, which correlated to the flexion transient motor transduction decline. Furthermore, we firstly reported that the impartial angiogenesis in the anterior and posterior parts of the cervical cord parenchyma may be the underlying mechanism of drastic blood flow decrease only at a flexed position.

Currently, prolonged extension and flexion, especially extension position are commonly recognized as deleterious activities for CSM patients (Milligan et al., 2019). Cervical extensions make the ligamentum flavum bulge inward, decrease the dorsal subarachnoid space up to 17% (Muhle et al., 1998b) and increase the Mühle stenosis grade (Muhle et al., 1998a). On the other hand, although cervical flexion increases the dorsal subarachnoid space at each level from C2 to C7 (Muhle et al., 1998b), it also increases the longitudinal strain of the cord and induces compression against the ventral spondylotic bar (Hattou et al., 2014). Briefly speaking, the spinal cords are more likely to suffer from circumferential compressions at cervical extension due to pincer effects, and more severe anterior compressions at cervical flexion. In our previous clinical studies (Qi et al., 2020; Yu et al., 2020), we reported that most patients had DSSEP deterioration upon extension and flexion. However, the lack of DMEP data prevented more in-depth examinations of the neurophysiological functions of CSM patients, because the somatosensory and motor conductions were carried out by different structures within the spinal cord (de Haan and Kalkman, 2001) and thus may present different characteristics upon dynamic neck positions. Here, we found that unlike DSSEPs, the DMEPs of both the CSM patients and rats deteriorated particularly upon cervical flexion. Ueta et al. (1992) investigated the effects of transient focal compression in four different directions on physiologic integrity and electrophysiological manifestations including MEPs, SEPs and spine-to-spine potentials in pigs. They noted that SEPs were lost first during posterior, circumferential, and lateral acute transient compressions, while MEPs were lost first during anterior compression in pigs, which coordinated with our current finding in both CSM patients and rat models that DSSEPs were affected upon both extension (circumferential compression) and flexion, while DMEPs were mainly upon flexion (anterior compression).

Dynamic MRI in this study revealed that the intramedullary T2WI intensity in CSM rats was the greatest upon flexion compared with that at neutral or extension positions, which was in line with other CSM clinical studies (Zhang et al., 2011; Zeitoun et al., 2015). While some authors assumed that cervical canal expansion at flexion permits better visualization of intramedullary hyperintensity on T2WI (Zhang et al., 2011; Zeitoun et al., 2015), others concluded that hyperintensities were more related to spinal cord ischemia (Weidauer et al., 2015). Further studies have reported that the pattern of intramedullary hyperintensity lesions is an important criterion (Chen et al., 2001; You et al., 2015). In this study, we found a significant hyperintense “pencil-like” lesion pattern on sagittal T2WIs and the so-called “snake eyes” or “owl eyes pattern” with hyperintense signal conversion on axial T2WIs of CSM rats upon flexion (see Figures 5D,H). These patterns have been frequently reported as important MRI characteristics of spinal cord infarction, indicating ischemia and partially symmetrical small infarcts in the anterior horns (Weidauer et al., 2002, 2015). Moreover, numerous studies have already demonstrated the impact of ischemia on the neurophysiological and electrophysiological function of the spinal cord (Shine et al., 2008). We thus assume that the dynamic neurophysiological changes of CSM rats may be related to the dynamic perfusion decrease in cervical cord upon flexion.

Laser Doppler flowmetry is a non-invasive method using the tissue backscattered light to qualitatively assess the blood flow rate, which makes it preferable for measuring microcirculatory alterations of the spinal cord at different neck positions (Yamada et al., 1998; Olive et al., 2002; Jing et al., 2018). We found a sharp reduction of SCBF upon flexion compared with neutral position in CSM rats at 4 wpi, the degree of which was significantly correlated with the RA-DMEP rather than the RA-DSSEP in CSM rats at a flexed neck position, suggesting the MEPs are more sensitive to ischemic changes compared with SSEPs. These findings are in general agreement with some other studies. Bennett examined the effect of focal spinal cord ischemia induced by segmental dorsal and ventral rhizotomy on MEPs and SSEPs in the cat spinal cord (Bennett, 1983). He observed that MEPs more sensitively reflected spinal cord ischemia than SSEPs but he did not measure SCBF. Fehlings et al. (1989) reported that MEP amplitudes rather than SSEP amplitudes were significantly correlated with SCBF and injury severity (compression forces) in clip compression spinal cord injury rat models. Shine et al. (2008) reported that MEPs disappeared prior to SSEPs after introducing ischemia by aortic cross clamping; moreover, early MEP disappearance suggested a poor neurological outcome in patients undergoing thoracoabdominal aortic aneurysm repair. These animals and clinical studies indicated that compared with SSEPs, MEPs were more easily affected by spinal cord perfusion status. However, in addition to the spinal cord ischemia, other factors may also contribute to the high sensitivity of DMEPs to cervical flexion. For example, the corticospinal tract and the anterior horn motor neuronal system are mainly located at the ventral aspect of the spinal cord. Gooding et al. (1975) used dog models of cervical vascular insufficiency that were derived via selective vascular ligation to show that vascular disruption caused demyelination, glial fibrosis, and necrosis, especially in the corticospinal tracts. Histological abnormalities in all dogs with combined compression and ischemia were more pronounced than in dogs with either compression or ischemia alone (Gooding et al., 1975). Thus, DMEP deterioration upon flexion in our CCSCI rat models was more likely to be due to the combinatorial effects of ischemia and some other factors including the direct neuronal compression.

To further investigate the underlying mechanisms of drastic blood perfusion loss upon flexion in CSM conditions, we performed histological examinations on the cervical cords from both the sham and CSM rats. Our previous study showed that 4 weeks of chronic compression led to a severe ischemia-hypoxia environment as demonstrated by increased HIF-1α expression, and decreased microvascular density in the overall spinal cord compression site as demonstrated by micro-CT images (Cheng et al., 2019, 2020, 2021). However, they could not explain the disparity between the perfusion loss upon cervical flexion and extension. The current study further investigated the distribution of CD31, VEGF and vWF expression in the anterior and posterior parts of the spinal cord after chronic compression. The CD31 immunostaining has been frequently used to assess the vascular density (Bianconi et al., 2020), while the VEGF and vWF have been reported as angiogenesis biomarkers in many diseases (Starke et al., 2011). Apart from the overall vascular changes, microvascular density assessed by CD31 were significantly increased in the anterior horn but decreased in the dorsal horn, and angiogenesis marked by vWF and VEGF were increased only in anterior horns in chronically compressed spinal cords. Morphologically, well-formed capillaries stained with CD31 were also mainly observed in the anterior horns, suggesting the microvessels in the anterior spinal parenchyma were functional and can lead to a better blood perfusion in the anterior compared to the posterior part of the spinal cord. At neutral and extension neck positions, because the compression on the anterior part of the spinal cord is milder, the blood perfusion of the whole spinal cord can be compensated by the relatively denser blood vessels in the anterior spinal cord parenchyma, making the gross SCBF less affected. Upon flexion, however, the whole spinal cord is subjected to significantly more severe ischemia and hypoxia because the compensatory perfusion in the front of spinal cord is blocked. The vascular distribution disparity in the anterior and posterior parts of the spinal cord is significantly correlated with the exacerbated ischemia and hypoxia of the whole spinal cord upon flexion as measured by our dynamic LDF, which further demonstrates the vascular redistribution in the spinal cord parenchyma could be an important mechanism underlying the drastic decrease of perfusion upon flexion position in CSM rats.

There are a number of limitations to the present study. Firstly, although our study demonstrated the correlation between the electrophysiological functional and blood perfusion changes, a causal relationship between the two has not been formally demonstrated. Nevertheless, it is likely that the dynamic neurophysiological changes can be also attributed to neuronal and axonal compression at dynamic neck positions. Secondly, the CSM rats’ cervical cords were compressed posteriorly, while the CSM patients were mainly suffer from anterior compression. It remains unclear whether a different direction of the implant in the spinal cord may lead to chronic compressive injury in a different spinal cord area or tract, thus resulting in different neurophysiological and hemodynamic performance.

## Conclusion

In conclusion, the data from the present clinical trial and animal experiment show that changes in the motor and somatosensory conductive function of the spinal cord, as assessed by DMEPs and DSSEPs present distinct characteristics at dynamic neck positions. The more severe motor conductive dysfunction compared to the somatosensory deficit upon neck flexion was significantly correlated with the dynamic spinal cord ischemia at flexion, which could be partly attributed to MEPs’ higher susceptibility to ischemia compared to SSEPs and the vascular redistribution in the spinal cord parenchyma of CSM rats. Such a notion might open a new possible path for medical treatment for CSM, as opposed to the current practice that relies heavily on surgical decompression. Immobilization, such as that achieved by the use of cervical collars to prevent neck flexion in CSM patients, or drug treatment aiming to improve spinal cord perfusion may be effective in preventing the progression of functional loss.

## Data Availability Statement

The original contributions presented in the study are included in the article/Supplementary Material, further inquiries can be directed to the corresponding authors.

## Ethics Statement

The studies involving human participants were reviewed and approved by institutional review board (IEC) for clinical and animal trials of The First Affiliated Hospital of Sun Yat-sen University. The patients/participants provided their written informed consent to participate in this study. The animal study was reviewed and approved by Animal Experimentation Ethics Committee and Institutional Animal Care and Use Committee (IACUC) at The First Affiliated Hospital of Sun Yat-sen University.

## Author Contributions

ZY, XC, and JC were involved in performing animal experiments, analysis and interpretation of data, as well as in drafting and revising the manuscript. XZ and XP contributed to the conception and design of the study, and gave final approval of the version to be published. ZH revised the article critically. SH, HH, SL, ZZ, FH, and BC were involved in acquisition of data. All authors read and approved the final manuscript.

## Conflict of Interest

The authors declare that the research was conducted in the absence of any commercial or financial relationships that could be construed as a potential conflict of interest.

## Publisher’s Note

All claims expressed in this article are solely those of the authors and do not necessarily represent those of their affiliated organizations, or those of the publisher, the editors and the reviewers. Any product that may be evaluated in this article, or claim that may be made by its manufacturer, is not guaranteed or endorsed by the publisher.

## Abbreviations

ASA: anterior spinal artery
BBB: Basso, Beattie, and Bresnahan scale
IPT: inclined plane test
CSM: cervical spondylotic myelopathy
DMEPs: dynamic motor evoked potentials
DSSEPs: dynamic somatosensory evoked potentials
HE: hematoxylin and eosin staining
LDF: laser Doppler flowmetry
MRI: magnetic resonance imaging
RA-DMEP: relative amplitude of dynamic motor evoked potential
RA-DSSEP: relative amplitudes of dynamic somatosensory evoked potential
R-SCBF: relative change in spinal cord blood flow
R-SO 2: relative change in spinal cord oxygen saturation
SCBF: spinal cord blood flow
T2WI: T2-weighted image.
