# Supplementary material for: Spinal Cord Parenchyma Vascular Redistribution Underlies Hemodynamic and Neurophysiological Changes at Dynamic Neck Positions in Cervical Spondylotic Myelopathy
Source: Front Neuroanat. 2021 Nov 23;15:729482. doi: 10.3389/fnana.2021.729482 (PMC8650056; doi:10.3389/fnana.2021.729482)
Supplement: Supplementary file 1 [file Data_Sheet_1.DOCX]

**Supplementary materials:**

Fig S1. The instant spinal cord blood flow and oxygen saturation level at 10 minutes after the compression or sham surgery.

The spinal cord blood flow (SCBF) (a) and oxygen saturations (SO_2_) (b) were measured by a laser Doppler flowmetry at the C5/6 segment of the cervical cords upon dynamic neck positions in Sham and CCSCI models. Both of the two parameters were similar between the Sham and CCSCI models at 10 min after implanting the compression material.


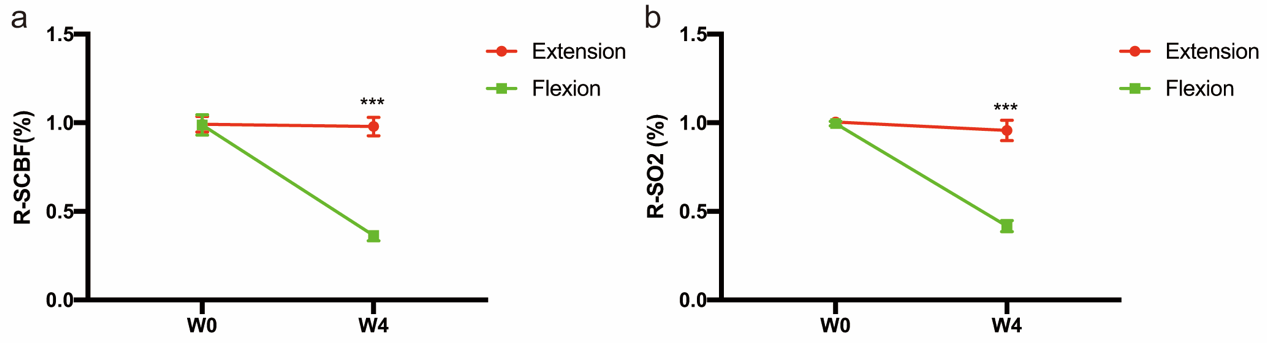


Fig S2. The relative change of spinal cord blood flow and oxygen saturations upon extension and flexion.

The relative change of spinal cord blood flow (R-SCBF) and oxygen saturation (R-SO_2_) upon extension or flexion at 10 minute- and at 4 week-post-injury were calculated by dividing each model's SCBF at extension or flexion by that at neutral position. The R-SCBF and R-SO_2_ at flexion was significantly lower than the that at extension (t-test, p<0.001).


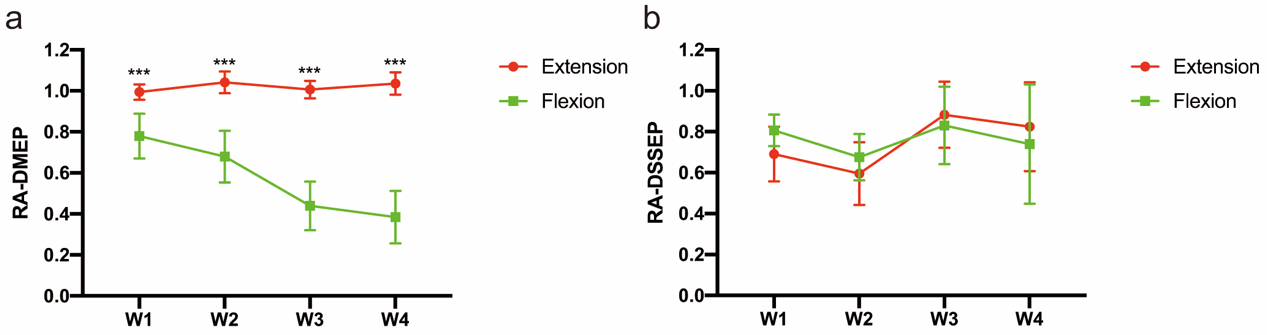


Fig S3. The relative amplitudes of DMEPs and DSSEPs upon extension and flexion.

The relative amplitudes of DMEPs (RA-DMEP) or DSSEPs (RA-DSSEP) were calculated by dividing each model's DMEP N1 or DSSEP N13 amplitude at extension or flexion by that at neutral position. The RA-DMEP upon flexion gradually decreased and were significantly lower than that upon extension at all time points after injury (t-test, p<0.001), while the RA-DSSEP upon extension and flexion did not vary significantly at all time points after injury.
